# An Innovative Injection Technique for Treating Edematous Fibrosclerotic Panniculopathy (Cellulite): A Prospective Multicenter Clinical Investigation

**DOI:** 10.1093/asjof/ojag137

**Published:** 2026-07-17

**Authors:** Elena Fasola, Andreea Boca, Giorgio Reggiardo, Vincenzo Nobile, Dhouha Dridi

## Abstract

Edematous fibrosclerotic panniculopathy (EFP) affects ∼80% to 98% of postpubertal women and has a significant impact on quality of life. Its unclear etiopathology and the multifactorial origin pose a significant challenge for its clinical management. This study aimed to evaluate the efficacy and the safety of a commercially available mixture of hyaluronic acid and amino acids in an alkaline solution. A prospective multicenter clinical trial was carried out on 46 women with EFP Stage I to early Stage IV. The improvement of cellulite severity was evaluated by the modified Clinician-Reported Photonumeric Cellulite Severity Scale (CR-PCSS) and the modified Patient-Reported PCSS (PR-PCSS) before the first (T1), the second (T2), and the third injection (T3) and after 3 (T4) and 6 (T5) months of treatment. The psychological impact of cellulite was measured using the Objectified Body Consciousness (OBC) scale. The PR-PCSS revealed that 95.3% of participants reported an improvement in their appearance. These positive outcomes remained consistent up to 6 months following the initial treatment. Similarly, the CR-PCSS showed that as early as 2 weeks after the first treatment, 76.7% of participants demonstrated objective improvement based on the Global Aesthetic Improvement Scale. These improvements also remained stable for up to 6 months. Self-esteem (OBC scale) was improved. No major adverse events were reported; minor bruising occurred as expected because of injections. Sunekos Cell 15 is a possibly effective and well-tolerated treatment for cellulite, particularly in cases with pronounced fibrosis, potentially offering a minimally invasive therapeutic option.

Level of Evidence: 4 (Therapeutic)

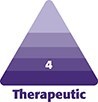

Edematous fibrosclerotic panniculopathy (EFP), commonly known as cellulite, is a localized metabolic disorder of the subcutaneous tissue characterized by progressive structural and functional alterations involving the dermis, the adipose lobules, and the fibrous septa.^1^ These changes are associated with impairment of the microcirculation and lymphatic drainage, leading to interstitial edema, tissue hypoxia, and low-grade inflammation processes that contribute to fibrosclerosis and remodeling of the extracellular matrix (ECM).^1,2^ Clinically, EFP manifests as an irregular “dimpled” or “orange-peel” skin surface, most commonly affecting the thighs, buttocks, and hips, with variable severity and impact on quality of life (QoL).^3^ Despite being a benign condition, it represents a major aesthetic concern and a frequent reason for seeking medical or cosmetic intervention. EFP is highly prevalent, affecting an estimated 80% to 98% of postpubertal women, likely reflecting a combination of hormonal influences, female-specific connective tissue architecture, and genetic and lifestyle-related factors.^1,4,5^ Despite numerous studies in literature, the etiopathology of EFP remains incompletely understood and is widely considered multifactorial.^1^ Current evidence suggests that a convergence of (1) structural alterations of the fibrous septa and ECM, (2) inflammatory and low-grade edematous changes, (3) morphological remodeling of adipose lobules and dermal architecture, and (4) biochemical changes in the subcutaneous tissue may collectively contribute to the development and progression of this condition.^2,6^ Lotti et al demonstrated that the precipitation of granular electrodense material in the dermal capillary walls and adjacent to the collagen and elastic fibers of the dermis is associated with swelling of the ground substance, which may lead to structural alterations of the fibers followed by sclerosis.^7^

The incomplete understanding of EFP pathophysiology represents a major limitation for evidence-based clinical management, contributing to heterogenous treatment responses and a persistent unmet need in routine practice, greatly impacting women's QoL. This context highlights the importance of continuous research aimed at identifying and targeting the multiple biological pathways implicated in cellulite and at developing strategies and ingredients specifically capable of addressing its structural, microcirculatory, inflammatory, and extracellular-matrix components.^8^ Such advancements would enable more personalized and effective treatments while reducing the burden of this common aesthetic condition and improving outcomes for affected women.

Against this background, we conducted this prospective observational clinical study to evaluate the safety and efficacy of a commercially available Class III injectable medical device (Sunekos Cell 15, Professional Dietetics S.p.A., Milano, Italy) in 46 women with EPF stage from I to early Stage IV. Efficacy was assessed primarily through patient-centered endpoints, including women's satisfaction and treatment tolerability to align clinical evaluation with real-world experience. In addition, we investigated the impact of the treatment on body perception recognizing that perceived aesthetic improvement and body image are clinically relevant outcomes in the management of cellulite. This integrated approach provides a more comprehensive characterization of treatment performance by combining objective, visually appreciable effects with the patient's perception of efficacy.^9^ Sunekos Cell 15 is formulated with 3 key components, each selected to deliver a targeted, specific action: (1) low molecular weight hyaluronic acid (200 kDa), which plays a crucial role in hydrating the extracellular space while enhancing the dermis's viscoelasticity and the mechanical properties of the ECM; (2) a blend of 6 amino acids (glycine, L-proline, L-lysine, L-leucine, L-valine, and L-alanine), which support hydration, maintain optimal pH buffering and osmolarity within the tissue microenvironment, and reduce hyaluronic acid degradation by inhibiting hyaluronidase activity; and (3) a carbonate-bicarbonate buffer (pH 8.2), which helps neutralize the acidosis typically associated with latent inflammation, a hallmark of both early and advanced stages of extracellular fibrotic pathology.^10-14^

## METHODS

### Trial Design and Ethics

The trial was a prospective multicenter study conducted at the Gyplast Medical Institute (Milan, Italy) and DEA Clinic (Cluj-Napoca, Romania) from March to November 2024. Participants attended 3 treatment visits scheduled at 14- or 21-day interval. Follow-up assessments were performed at 3 and 6 months after the first treatment session.

At baseline, all participants underwent clinical and anthropometric evaluations. Clinical outcomes were assessed by independent physicians who were not involved in the data analysis, ensuring a clear separation between outcome evaluation and statistical processing and minimizing the risk of assessment bias.

Informed consent was obtained following a thorough medical consultation about the study and before the initiation of the treatment protocol. The study was conducted in accordance with Good Clinical Practice, the ethical principles outlined in the Declaration of Helsinki, and applicable regulations governing observational studies.

### Participants

Female patients aged 18 to 40 years with Stage I, II, III, or early Stage IV cellulite, as classified by the Curri scale, were deemed eligible for inclusion in the study.^15^ The eligible participants were enrolled if they met the following inclusion criteria: no known allergies to the components of the study product, no previous cellulite treatments, no autoimmune diseases, and availability for follow-up visits through the entire study period. Exclusion criteria included: sensitivity to any component of the product; ongoing inflammatory or infectious conditions in the treatment area (eg, eczema, dermatitis, fungal infections, herpes, and bacterial infections); current anti-inflammatory or anticoagulant therapy; autoimmune diseases affecting soft tissue; active cancer treatment; major systemic diseases; a predisposition to keloid formation; unrealistic treatment expectations; being obese; and advanced, late-stage Grade 4 cellulite, characterized as irreversible and nearly completely sclerotic.

### Interventions

**Figure 1.**
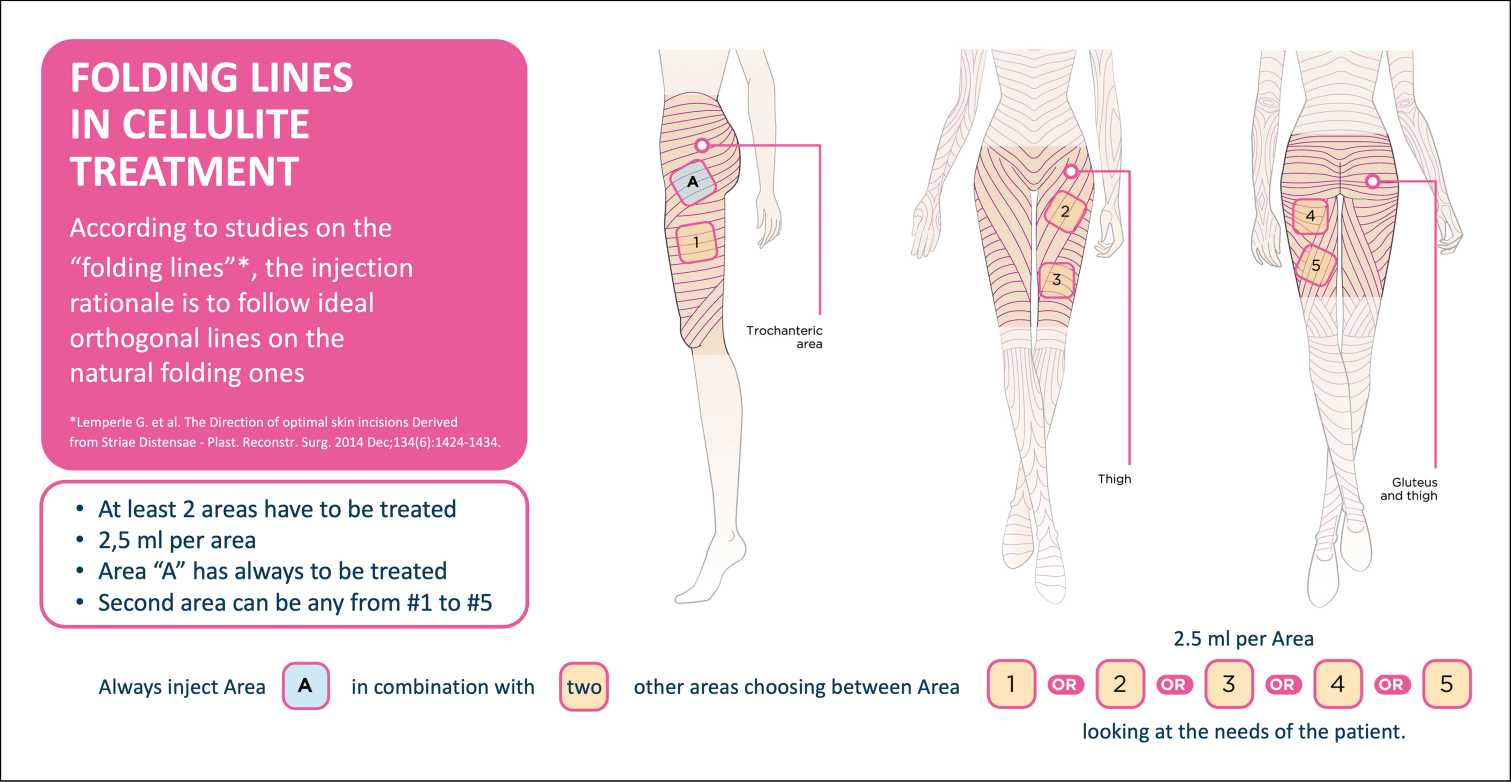
Across tension line technique (ATLT). The treatment protocol consistently included Site A, whereas 2 additional injection sites were selected from Regions 1 to 5, according to the ATLT and Langer's Lines.

The intervention consisted of the injection of a mixture of low molecular weight hyaluronic acid (200 kDa) and 6 amino acids (glycine, L-proline, L-lysine, L-leucine, L-valine, L-alanine) reconstituted in an alkaline (carbonate and bicarbonate salts) solution (Sunekos Cell 15). The injection was performed by a 27 or 22 G (40 or 70 mm) cannula at 3 different sites in the trochanteric-gluteal-femoral area according to the across tension line technique (Figure 1).^16,17^ The product was injected in a fan-shaped pattern. The fan consisted of 3 linear retrograde injection passes, each separated by ∼45°. The procedure began with a first linear pass; then rather than fully withdrawing the cannula between deposits, the cannula was redirected and repositioned to create the subsequent passes before reemerging through the same entry point. The injection procedure is shown in the Video. Each site was injected with 2.5 mL of product (7.5 mL of product per side). The treatment was repeated 3 times (at T1, T2, and T3) at intervals of 14 to 21 days.

### Outcomes and Endpoints

The study aimed to evaluate the safety and efficacy of Sunekos Cell 15 for the treatment of EFP by assessing treatment tolerance and patient satisfaction. Satisfaction and treatment tolerability were assessed using a 5-point Likert scale, with response options ranging from 1 = very satisfied, 2 = satisfied, 3 = neither satisfied nor dissatisfied, 4 = dissatisfied to 5 = very dissatisfied.

Cellulite severity was evaluated using a modified version of the Clinician-Reported Photonumeric Cellulite Severity Scale (CR-PCSS) and the Patient-Reported PCSS (PR-PCSS), based on standardized imaging assessments conducted at baseline (T1), T2, and T3.^18^ The scales were adapted by incorporating standardized reference images representing varying grades of cellulite severity in both the buttock and thigh regions. These reference images facilitated the comparison of treatment outcomes in accordance with the Global Aesthetic Improvement Scale (GAIS). Originally developed for aesthetic clinical trials, the GAIS was adapted to quantify changes in cellulite appearance from both the clinician's perspective (investigator-GAIS, I-GAIS) and the patient's perspective (participant-GAIS, S-GAIS).^19-21^ Participants self-assessed their perceived aesthetic improvement using a 5-point Likert scale: 1 = exceptionally improved, 2 = much improved, 3 = improved, 4 = no change, and 5 = worsened.

Self-esteem was assessed at baseline, T4 and T5 with the Objectified Body Consciousness (OBC) scale which evaluates women's body perception.^22^ OBC consists of 3 components: (1) body surveillance, (2) internalization of cultural body standards, and (3) beliefs about appearance control.^23,24^ Women respond on a scale of 1 to 7 where 1 means strongly disagree and 7 means strongly agree. Higher OBC scores indicate a more negative body experience.

### Statistical Analysis

Data were analyzed by an independent statistician. A frequency analysis was performed for all variables included in the study. Continuous variables were summarized using the mean and standard deviation (SD), whereas categorical variables were reported as absolute counts and percentages. Statistical associations between categorical variables were evaluated using the χ^2^ test or Fisher's exact test, as appropriate. To explore potential age-related trends in treatment response, a univariate linear regression analysis was conducted, assessing changes in PR-PCSS scores from baseline in relation to participants’ age. Beta coefficients from the regression model were used to estimate the extent of PR-PCSS variation associated with age. Data analyses were performed using IBM SPSS Statistics Ver. 29 (IBM Corp., Armonk, NY).

## RESULTS

### Participant Characteristics

Twenty women were recruited at Gyplast Medical Institute and 26 women at DEA Clinic in Romania for a total of 46 participants. All participants received the first treatment session, whereas only 44 participants proceeded to the second and third sessions. One participant was lost to follow-up after the third treatment session. Participants who did not complete the study were excluded from the demographic and efficacy analyses, whereas all participants who received at least 1 treatment were included in the safety analysis set. Consequently, the efficacy analysis set included 43 (*n* = 43) participants, whereas the safety analysis set included 44 participants (*n* = 44).

Briefly, the participants’ mean age was 32.8 ± 5.5 years (18-40 years), the mean BMI was 22.5 ± 3.6 kg/m^2^, smokers were 37.2%, nonsmokers were 62.8%, and 72.1% reported occasional/regular alcohol consumption. Among the study participants, 32.2% were taking progestin or estroprogestin hormone therapy. Demographic characteristics are reported in Table 1.

**Table 1.** Demographic Characteristics

Characteristics	Value	Units
Age	32.8 ± 5.5	Years
BMI	22.5 ± 3.6	kg/m^2^
Smoke		
Never smoker	62.8 (27/43)	% (*n*)
Current smoker	37.2 (16/43)	% (*n*)
Ex-smoker	0 (0/43)	% (*n*)
Alcohol consumption		
Yes	72.1 (31/43)	% (*n*)
No	27.9 (12/43)	% (*n*)
Hormonal therapy		
Yes	23.2 (10/43)	% (*n*)
No	76.8 (33/43)	% (*n*)

Continuous data are expressed as the mean ± standard deviation. Categorical data are expressed as percentages and counts.

### Likert Scale Results: Treatment Tolerability

Forty-four participants completed all 3 treatment sessions according to the study protocol and responded to the 5-point Likert scale for treatment tolerability. The remaining 2 participants did not complete the 3 sessions, were lost to follow-up at T2, and were excluded from the respective results. At baseline, after the first treatment session, 73.9% of the 46 enrolled participants (95% CI, 58.8-85.7) reported experiencing no pain during the procedure. Eleven participants (23.9%; 95% CI, 12.5-38.7) were neither satisfied nor dissatisfied, and only one participant (2.1%; 95% CI, 0.05-11.5) reported experiencing pain during the treatment (Table 2).

**Table 2.** Treatment Tolerability

Likert scale	T1	T2	T3
Very satisfied	34.8 (16/46)	34.0 (15/44)	43.1 (19/44)
Satisfied	39.1 (18/46)	34.0 (15/44)	31.8 (14/44)
Neither satisfied nor dissatisfied	23.9 (11/46)	13.6 (6/44)	20.4 (9/44)
Dissatisfied	2.1 (1/46)	15.9 (7/44)	4.5 (2/44)
Very dissatisfied	0 (0/46)	2.2 (1/44)	0 (0/44)

At T2, 68.1% of participants (95% CI, 52.4-81.3) reported experiencing no pain during the treatment. Six participants (13.6%; 95% CI, 5.1-27.3) were neither satisfied nor dissatisfied, whereas 7 participants (15.9%; 95% CI, 6.6-30.0) reported experiencing pain.

At T3, the majority of participants (74.9%; 95% CI, 59.6-86.8) reported being satisfied and experiencing no pain, 20.4% were neither satisfied nor dissatisfied (95% CI, 9.8-35.3), and only 2 participants (4.5%; 95% CI, 0.5-15.4) reported experiencing pain.

Throughout the study, no major side effects were reported by any of the participants from the 2 centers. Overall, the treatment was well tolerated, and any reactions observed were limited to mild, transient effects typically associated with injectable procedures.

### CR-PCSS and PR-PCSS Results

#### Clinician-Reported Photonumeric Cellulite Severity Scale

At T2, over 3 quarters of the study participants (76.7%; 95% CI, 61.3-88.2) showed objective improvement based on the physician's assessment using the GAIS scale. Nine out of 42 women (21.4%) showed no change at reassessment, and no cases of worsening were observed (Table 3).

**Table 3.** Clinician-Reported Photonumeric Cellulite Severity Scale (CR-PCSS) and Patient-Reported Photonumeric Cellulite Severity Scale (PR-PCSS) Scoring

	T2	T4	T5
PR-PCSS			
Exceptionally improved	18.2 (8/44)	28.5 (12/42)	23.2 (10/43)
Much improved	52.3 (23/44)	38.1 (16/42)	34.8 (15/43)
Improved	22.7 (10/44)	30.9 (13/42)	37.2 (16/43)
Unchanged	6.8 (3/44)	2.3 (1/42)	4.6 (2/43)
Worsening	0 (0/44)	0 (0/42)	0 (0/43)
CR-PCSS			
Exceptionally improved	30.2 (13/43)	41.4 (17/41)	28.5 (12/42)
Much improved	48.8 (21/43)	26.8 (11/41)	45.2 (19/42)
Improved	20.9 (9/43)	31.7 (13/41)	23.8 (10/42)
Unchanged	0 (0/43)	0 (0/41)	2.3 (1/42)
Worsening	0 (0/43)	0 (0/41)	0 (0/42)

The number of participants is variable, because of missing data. Data are reported as percentage and counts.

At T4, 68.2% of study participants (28/41; 95% CI, 51.9-81.9) showed objective improvement. Thirteen out of 41 women (31.8%) showed no change at reassessment, and no cases of worsening were reported.

Six months after the start of treatment (T5), 73.8% of study participants (95% CI, 57.9-86.1) showed objective improvement. Ten women (23.8%) showed no change, and 1 woman (2.4%) exhibited worsening. Results were statistically significant (*P* < .001).

**Figure 2.**
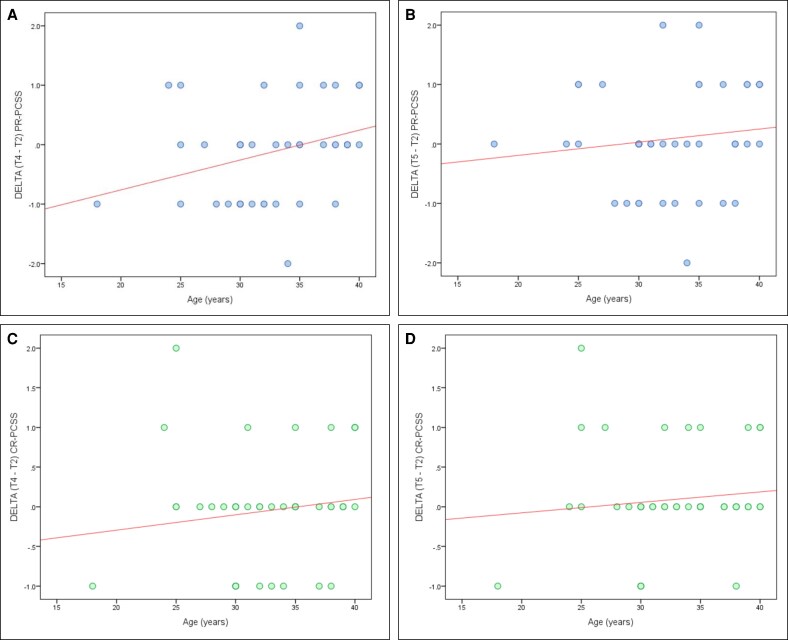
Univariate linear regression analysis. (A) Trend of change in Patient-Reported Photonumeric Cellulite Severity Scale (PR-PCSS) from T2 to T4 by patient’s age, expressed in years. (B) Trend of change in PR-PCSS from T2 to T5 by patient’s age, expressed in years. (C) Trend of change in Clinician-Reported Photonumeric Cellulite Severity Scale (CR-PCSS) from T2 to T4 by patient’s age, expressed in years. (D) Trend of change in CR-PCSS from T2 to T5 by patient’s age, expressed in years.

Univariate linear regression analysis of the PR-PCSS and CR-PCSS scores revealed that patients older than 30 years reported greater improvement, with higher scores observed from T2 to T4. However, this perceived improvement tended to diminish at the 6-month follow-up (T5) compared with younger participants. Indeed, in the linear regression analysis examining the relationship between the change in PR-PCSS from baseline to Visit T4, the regression coefficient of 0.05 indicated that the PR-PCSS delta score (T4-T2) increased by 0.05 for each additional year of age (Figure 2).

#### Patient-Reported Photonumeric Cellulite Severity Scale

At T2, 41 of the enrolled participants (95.3%; 95% CI, 84.1-99.4) reported a statistically significant improvement in cellulite severity according to GAIS. Specifically, 8 participants (18.6%; 95% CI, 8.3-33.4) reported exceptional improvement, 23 participants (53.5%; 95% CI, 37.6-68.8) reported much improvement, and 10 participants (23.2%; 95% CI, 11.7-38.6) reported some improvement. Three participants reported no change, and no cases of worsening were observed (Table 3).

At T4, 12 out of 42 participants (28.5%; 95% CI, 15.7-44.5) reported exceptional improvement, 16 participants (38.1%; 95% CI, 23.5-54.3) reported much improvement, and 13 participants (30.9%; 95% CI, 17.6-47.0) reported some improvement. One participant (2.3%; 95% CI, 0.06-12.5) reported no change, and no cases of worsening were observed. Additionally, 1 participant did not respond (missing data).

**Figure 3.**
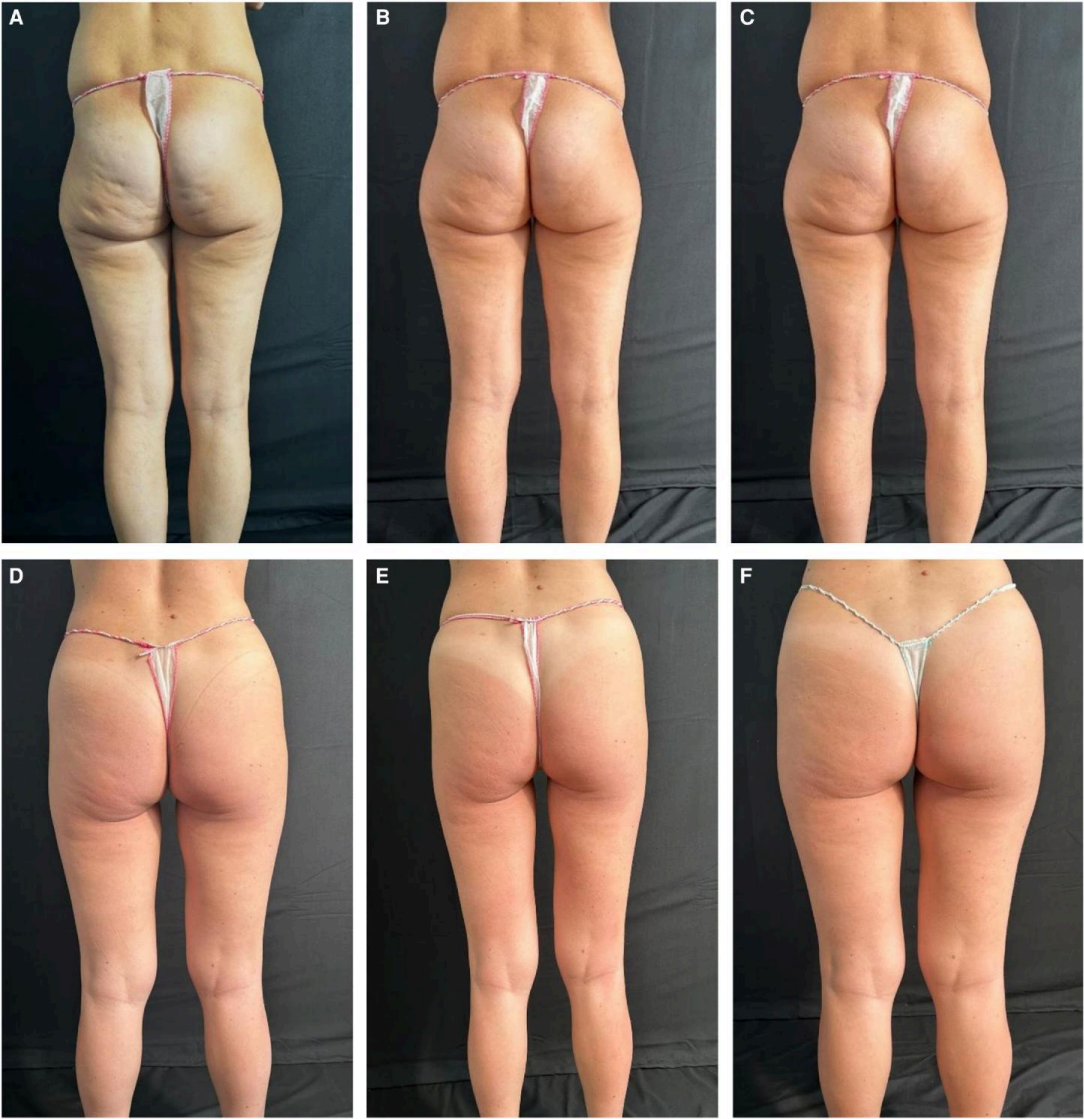
Improvement of edematous fibrosclerotic panniculopathy signs. (A) Stage III (a 37-year-old patient) at baseline. (B) Stage III (a 37-year-old patient) at T4 (3 months after the first treatment). (C) Stage III (a 37-year-old patient) at T5 (6 months after the first treatment). (D) Stage II (a 24-year-old patient) at baseline. (E) Stage II (a 24-year-old patient) at T4 (3 months after the first treatment). (F) Stage II (a 24-year-old patient) at T5 (6 months after the first treatment).

**Figure 4.**
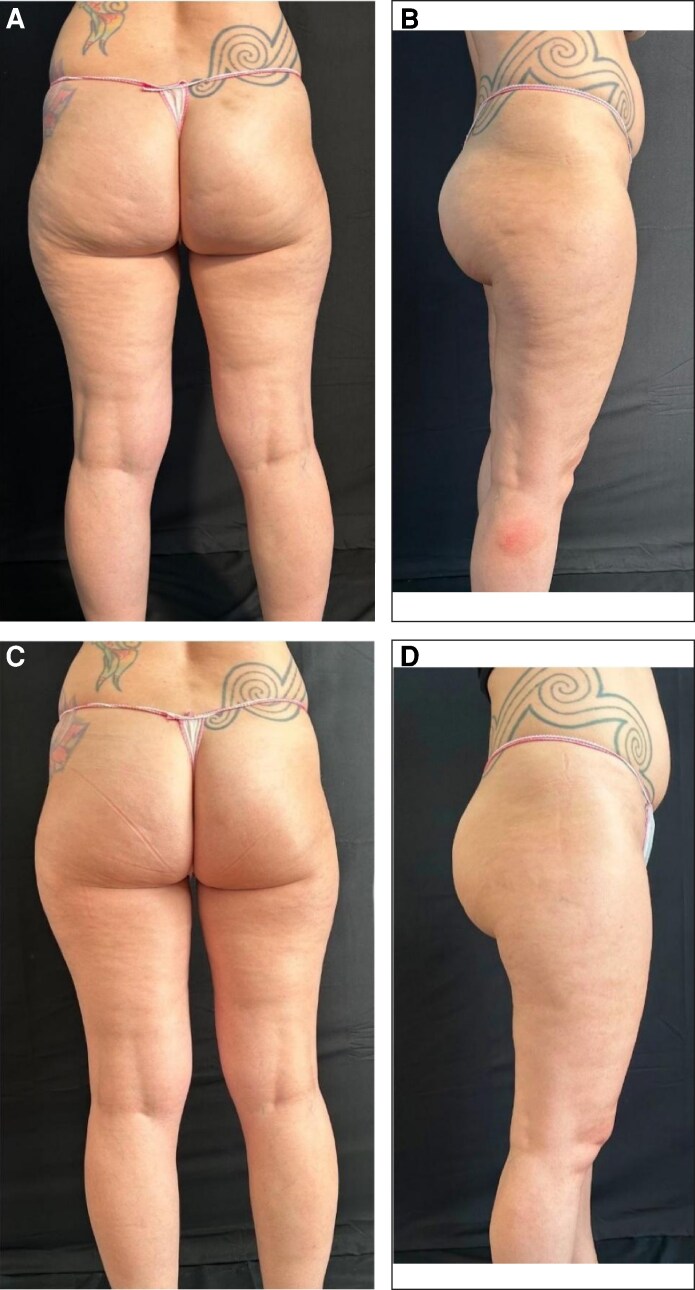
Improvement of edematous fibrosclerotic panniculopathy signs. (A) Early Stage IV (a 40-year-old patient) at baseline, frontal view. (B) Early Stage IV (a 40-year-old patient) at baseline, lateral view. (C) Early Stage IV (a 40-year-old patient) at T5 (6 months after the first treatment), frontal view. (D) Early Stage IV (a 40-year-old patient) at T5 (6 months after the first treatment), lateral view.

Six months after the end of treatment (T5), 10 out of 43 participants (23.2%; 95% CI, 11.7-38.6) reported exceptional improvement, 15 participants (34.8%; 95% CI, 21.0-50.9) reported much improvement, and 16 participants (37.2%; 95% CI, 22.9-53.2) reported some improvement. Two participants (4.6%; 95% CI, 0.5-15.8) reported no change, and no cases of worsening were observed. Results were always statistically significant (*P* < .001). Figures 3 and 4 show the patients who reported the most pronounced positive effects following the treatment.

#### The Objectified Body Consciousness (OBC) Scale Results

**Figure 5.**
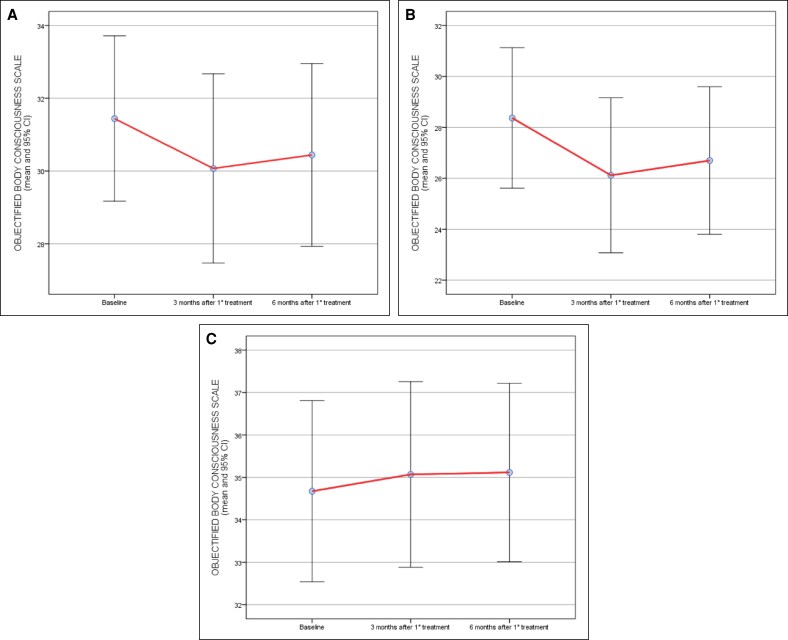
(A) Surveillance scale chart. (B) Body shame scale chart. (C) Control scale chart.

Both the surveillance and the body shame subscale scores of the OBC scale (Figure 5A, B) were statistically significantly (*P* < .001) reduced observed from baseline to 3-month follow-up, with a slight increase at 6 months.

The appearance control beliefs subscale showed a slight increase from baseline to 3 months of follow-up and then remained stable until the sixth month of follow-up (*P* < .001; Figure 5C).

## DISCUSSION

Our results confirmed the safety and efficacy of the investigational product (Sunekos Cell 15) in the treatment of Grade I to early Grade IV cellulite. The novelty of this treatment may reside in its specific action on cellulite characterized by fibrosis. These findings support the hypothesis that targeting ECM remodeling could be a key determinant for achieving clinically satisfactory outcomes in cellulite management and may plausibly translate into improvements in the self-esteem of women with this condition.

Bass et al, in a narrative review on the epidemiology and treatments of cellulite, concluded that there is a lack of clarity regarding the etiology of cellulite. However, clinical evidence suggests that fibrous septa play a central role in its pathophysiology.^3,25^ In fact, Kruglikov and Scherer suggested that the gluteofemoral white adipose tissue (gfWAT) depot creates conditions for the local activation of TLR4 receptors. This makes the tissue more sensitive to low doses of endogenous lipopolysaccharides present in gfWAT, which induces low-grade inflammation and promotes the irregular accumulation of collagen, as well as the development of fibrosis in this fat depot.^26^ For this reason, several studies have focused on investigating various techniques to treat the fibrosis characteristic of this condition, as it complicates the resolution of cellulite and impedes the achievement of optimal results. In fact, our findings demonstrated significant improvement in both the clinical picture and self-perception among women aged 35 to 40 years with more fibrotic cellulite compared with younger women with more edematous cellulite. However, this improvement, especially the CR-PCSS, was found to decrease at the 6-month follow-up, suggesting the value of implementing an additional 3-month treatment cycle or a maintenance protocol. This plan should be established during the initial assessment of the participants, before starting the first treatment cycle, to ensure long-lasting results and sustained satisfaction over time.

Foppiani et al conducted a recent systematic review and meta-analysis to assess the safety and efficacy of *Clostridium histolyticum* collagenase (CCH) and tissue subcision for the treatment of cellulite. The results concluded that both treatments appear to be effective. However, upon evaluating adverse outcomes between the 2 modalities, subcision showed a higher incidence of bruising, albeit with similar rates of induration compared with CCH injection. Conversely, the CCH injection group exhibited a higher propensity for pain requiring analgesia and notably showed increased instances of skin discoloration compared with the subcision group.^27^

Rossi and Vergnanini explained in a literature review the mechanisms behind the changes in the ECM that can explain the origin of cellulite. They describe how various factors, such as age, topographical changes, hormonal fluctuations, the use of drugs like corticosteroids and pregnancy, hyperthyroidism, diabetes, and free radicals, can affect ECM proteoglycans.^2^ The review highlights how fibroblast alterations, primarily triggered by estrogen, lead to structural changes in glycosaminoglycans within the dermis and perivascular connective tissue.^2,28^ This results in hyperpolymerization, which increases their hydrophilicity and interstitial osmotic pressure, leading to edema. The increase in viscosity causes cellular changes, compresses blood vessels, and ultimately leads to tissue hypoxia.^1,2,28^

Hypoxia alters aerobic glucose metabolism, leading to increased lactic acid production, which activates proline hydroxylase and enhances collagen production. Inflammation further accelerates collagen synthesis, resulting in the formation of incomplete, dysfunctional proteins that cannot maintain normal tissue functions or support connective tissue. Glycosaminoglycans maintain osmotic pressure, whereas proteoglycans are vital for collagen production and the reconstruction of the ECM.^2,29,30^

Our results may be consistent with the mechanism of action of the investigational product, potentially supporting its role in increasing the hydration of the extracellular space and in improving dermal viscoelasticity and the mechanical properties of the ECM. This effects could, in turn, favor collagen and elastin synthesis, help mitigate septal fibrosis, enhance tissue elasticity, and thereby contribute to improvement of skin appearance. These findings suggest that Sunekos Cell 15 may be particularly beneficial in treating moderate to advanced stages of cellulite (Stage II and early Stage IV), offering significant improvements for women who are typically considered untreatable, thereby raising treatment expectations. Indeed, Sunekos Cell 15 is an injectable medical device with therapeutic indication. No adverse events were reported except for bruises in some cases, which were already expected given the injective nature of the treatment. Sunekos Cell 15 may represent then a clinically valuable option for managing fibrosis related features of EFP while avoiding procedures such as subcision and other more traumatic interventions. In susceptible patients, these approaches may increase local tissue trauma and inflammation, potentially aggravating the underlying pathological cascade, exacerbate the condition, and perpetuate the pathological and vicious cycle. In this context, Sunekos Cell 15 offers a gentler strategy aimed at improving the structural component of cellulite with limited discomfort and a relatively rapid onset of visible improvement. Overall, the observed aesthetic benefits, combined with good tolerability, suggest a meaningful positive impact on patient-reported outcomes and QoL.

However, the present study has some limitations. Key variables that could influence treatment outcomes, such as smoking and alcohol consumption, were not accounted for in the data analysis. Additionally, because fibrosis appears to be a primary target of Sunekos Cell 15 treatment, further research could explore the relationship between fibrosis stage, age, and treatment response. The relatively small sample size could reduce the statistical power, whereas the relatively short duration of follow-up may be insufficient to fully capture the longer-term stability of the observed clinical effects. Despite these limitations, our study has several strengths. The fact that these results are derived from linear regression analysis involving only 40 women highlights the importance and value of the findings, despite some variability. Furthermore, the trend of lower improvement in CR-PCSS compared with PR-PCSS in the regression analyses demonstrates the objectivity of the data. The clinician's assessment is usually more critical and objective than the patient's self-assessment. Although the patient's self-assessment remains important, it is inherently subjective. Our goal, however, is to achieve a satisfactory outcome from both a clinical and aesthetic perspective, with a positive impact on the woman's QoL.

## CONCLUSIONS

The investigational product, Sunekos Cell 15, demonstrated an overall favorable efficacy and tolerability profile for the treatment of cellulite, with the greatest benefit observed in Stage II, Stage III, and early Stage IV, in which the fibrous component is typically more prominent. Conversely, the treatment appeared less effective in cases of early stage cellulite, where fluid retention and microcirculatory alterations may play a more central role than fibrosis. Although the available data are promising, further research is needed to assess the long-term effects.

## Data Availability

The data presented in this study are available on request from the corresponding author. The data are not publicly available since they are the property of the sponsor of the study (Professional Dietetics S.p.A., Milano, Italy).
